# Leprosy and its impact on the quality of life of people with physical disabilities: a scoping review

**DOI:** 10.1590/0034-7167-2023-0101

**Published:** 2024-07-15

**Authors:** Douglas Moreira de Araujo, Elisa Camila de Souza e Silva, Helen Vaz da Silva Gomes, Fábio da Costa Carbogim, Gesner Francisco Xavier, Angélica da Conceição Oliveira Coelho

**Affiliations:** I Universidade Federal de Juiz de Fora. Juiz de Fora, Minas Gerais, Brazil; II Universidade Federal de Minas Gerais. Belo Horizonte, Minas Gerais, Brazil

**Keywords:** Leprosy, Mycobacterium Leprae, Physically Disabled, People with Disability, Quality of Life, Lepra, Mycobacterium Leprae, Personas con Discapacidad Física, Personas con Discapacidad, Calidad de Vida

## Abstract

**Objectives:**

to identify and synthesize, from the literature, the impacts of physical disability caused by leprosy on the quality of life of individuals receiving care within the Health Care Network.

**Methods:**

this is a scoping review conducted following the JBI recommendations. The databases used in the search included the Medical Literature Analysis and Retrieval System Online, Cochrane Library, Web of Science, Lilacs, Cumulative Index to Nursing and Allied Health Literature, Scopus, Embase, Leprosy Information Services, and Google Scholar.

**Results:**

1690 documents were identified, of which 36 were included in the review. Physical disability caused by leprosy affects the quality of life in the areas of daily activities, socioeconomic aspects, psychological well-being, pain, and overall well-being.

**Conclusions:**

we identified the impairments caused by physical disability in the quality of life, highlighting the need for prevention, promotion, and rehabilitation actions, such as screening, case management, and health education.

## RESUMEN

**Objetivos:**

identificar y sintetizar, a partir de la literatura, los impactos de la discapacidad física causada por la lepra en la calidad de vida de los individuos atendidos en la Red de Atención a la Salud.

**Métodos:**

se trata de una revisión de alcance, realizada de acuerdo con las recomendaciones del JBI. Las bases de datos utilizadas en la búsqueda fueron el Sistema de Análisis y Recuperación de Literatura Médica en Línea, la Biblioteca Cochrane, *Web of Science*, Lilacs, el Índice Cumulativo de Literatura en Enfermería y Ciencias Afines, *Scopus, Embase, Leprosy Information Services* y *Google Scholar*.

**Resultados:**

se identificaron 1690 documentos, de los cuales 36 fueron parte de la revisión. La discapacidad física causada por la lepra afecta la calidad de vida en términos de actividades de la vida diaria, socioeconómicas, psicológicas, dolor y bienestar general.

**Conclusiones:**

se identificaron los compromisos causados por la discapacidad física en la calidad de vida, indicando la necesidad de acciones de prevención, promoción y rehabilitación, como el seguimiento, el acompañamiento de los casos y la educación en salud.

## INTRODUCTION

Leprosy is a tropical, neglected disease caused by Mycobacterium leprae, primarily affecting the skin and peripheral nerves, with a risk of progressing to physical disabilities (PD)^(1)^. Inadequate management of leprosy, along with late diagnosis and treatment, contributes to worse clinical outcomes^(2)^. In this context, the importance of healthcare professionals, especially nursing, being prepared to address this condition is emphasized, as nursing represents the largest number of professionals in the Health Care Network^(3)^.

In 2021, over 140,000 new cases were reported worldwide, with India leading in the absolute number of cases, followed by Brazil. Regarding the degree of physical disability, there were over 7,000 cases diagnosed with Physical Disability Grade 2 (PDG2)^(1)^. In Brazil, in 2022, 1,449 people were diagnosed with PDG2, and 4,041 with PDG1, corresponding to 11.6% and 32.4% of cases, respectively. It is noteworthy that the Brazilian states of Mato Grosso and Tocantins have the highest detection rates of new cases of the disease^(4)^. Early detection halts the progression of leprosy, preventing physical impairment and, consequently, social, emotional, and psychological harm, as well as deteriorated levels of quality of life^(5)^. According to the World Health Organization (WHO), quality of life is “the individual’s perception of their position in life, in the context of the culture and value systems in which they live and in relation to their goals, expectations, standards, and concerns”^(6)^.

In the health field, the assessment of quality of life is generally captured by instruments with appropriate psychometric properties that allow for the detection of physical, emotional, and social changes^(5-6)^. In leprosy literature, a review identified that the most frequently used instruments to assess quality of life are the WHOQOL-bref and the Medical Outcomes Study 36-Item Short-Form Health Survey (SF-36)^(7)^. This study also highlighted that the worst level of quality of life is associated with physical impairment, leprosy reactions, physical disabilities, neuropathic pain, and stigma^(7)^.

On the other hand, improving the quality of care for this population may involve actions of self-care, reducing sequelae, emotional and social impacts, and engagement in treatment and rehabilitation^(8-9)^. In this sense, detecting convergence between the disease’s sequels and aspects of quality of life can contribute to directing health actions. Despite the scientific knowledge about the physical and social damages related to leprosy being extensively studied, there is still a scarcity of scientific evidence mappings on physical disabilities and impacts on the quality of life of this population^(7-10)^.

Therefore, review studies on the quality of life of leprosy patients with physical disabilities are justified by the need to contribute to policies focused on health prevention and promotion, aiming to reduce the damages caused by the disease. Additionally, this can enable healthcare professionals to seek effective treatment alternatives and interventions for each case.

## OBJECTIVES

To identify and synthesize, from the literature, the impacts of physical disability caused by leprosy on the quality of life of individuals receiving care within the Health Care Network.

## METHODS

### Study Design

This study employs a scoping review based on the Joanna Briggs Institute (JBI). methodology, aiming to comprehensively cover the scientific literature for broad and inclusive results^(11)^. The study adhered to five methodological steps: 1) identification of the guiding question; 2) identification of relevant studies; 3) selection of studies; 4) mapping of information; 5) grouping, summarizing, and reporting results^(12)^. It is crucial to note that the concept of quality of life from the World Health Organization (WHO)^(6)^ was adopted for this review.

### Identification of the Guiding Question

The review aimed to address the following question formulated using the PCC acronym (Population, Concept, and Context): “What is the impact of physical disability on the quality of life of individuals affected by leprosy attended in the Health Care Network?”. In this framework, P (population) was defined as individuals affected by leprosy, C (concept) as the impact of physical disability on the quality of life, and C (context) as all levels of healthcare. The protocol for this review was registered on the Open Science Framework with the DOI 10.17605/OSF.IO/ZB4WR.

### Identification of Relevant Studies

An initial search was conducted on the Medical Literature Analysis and Retrieval System Online (MEDLINE) via PubMed to identify studies analyzing the impact on the quality of life of patients who developed physical disabilities due to leprosy. The descriptors “Leprosy”, “Hansen Disease”, “Disabled Persons”, “People with Disabilities”, and their possible synonyms were used, following the DeCS/MeSH platform, and the Boolean operators “AND” and “OR”. It’s worth noting that the descriptors “Health Care Levels” and “Quality of Life” were not used to avoid limiting search results.

The search strategy was adapted according to the specificity of each database (Chart 1): MEDLINE (PubMed), Cochrane Library, Web of Science, Latin American and Caribbean Health Sciences Literature (Lilacs) via the Virtual Health Library (BVS), Cumulative Index to Nursing and Allied Health Literature (CINAHL), Scopus, and Embase. Gray literature sources were explored, including Leprosy Information Services (INFOLEP) and Google Scholar, up to their first five pages. Studies published in English, Portuguese, and Spanish until August 30, 2023, were considered.

**Chart 1:** Search strategy according to the database

Database	Search Strategy
*Medline via Pubmed*	((“Leprosy”[Mesh] OR (Hansen’s Disease) OR (Hansen Disease) OR (Hansen^*^) OR (Lepr^*^)) OR (“Mycobacterium leprae”[Mesh] OR (Mycobacterium leprae))) AND (“Disabled Persons”[Mesh] OR (Disabled Person) OR (Person, Disabled) OR (Persons, Disabled) OR (Handicapped) OR (People with Disabilities) OR (Disabilities, People with) OR (People with Disability) OR (Persons with Disabilities) OR (Disabilities, Persons with) OR (Disability, Persons with) OR (Persons with Disability) OR (Physically Handicapped) OR (Handicapped, Physically) OR (Physically Disabled) OR (Disabled, Physically) OR (Physically Challenged))
*Cochrane*	*(Leprosy OR “Hansen Disease” OR “Mycobacterium leprae”) AND (“Disabled Persons” OR “Disabled Person” OR “Functional disability” OR “Functional disabilities”)*
*EMBASE*	*(‘leprosy’/exp OR leprosy OR ‘mycobacterium leprae’/exp OR ‘mycobacterium leprae’) AND (‘disabled persons’/exp OR ‘disabled persons’ OR ((‘disabled’/exp OR disabled) AND (‘persons’/exp OR persons)) OR ‘disabled person’/exp OR ‘disabled person’ OR ‘functional disability’/exp OR ‘functional disability’ OR ‘functional disabilities’)*
*CINAHL*	*(Leprosy OR “Hansen Disease” OR “Mycobacterium leprae”) AND (“Disabled Persons” OR “Disabled Person” OR “Functional disability” OR “Functional disabilities”)*
Lilacs via BVS	*(TW: Hanseníase OR “Doença de Hansen” OR Lepra OR “Hansen Disease” OR “Hansen’s Disease” OR “Enfermedad de Hansen” OR “Mal de Hansen” OR “Mycobacterium leprae” OR “Bacilo da Hanseníase” OR “Bacilo de Hansen” OR “Mycobacterium leprae” OR “Bacilo de Hansen” OR “Bacilo de la Hanseniasis” OR “Bacilo de la Lepra”) AND (TW: “Deficiência Física” OR “Deficiências Físicas” OR “Deficiente Físico” OR “Limitação Física” OR “Pessoa com Deficiência Fisica” OR “Pessoa com Desvantagem” OR “Pessoa com Incapacidade” OR “Pessoa com Incapacidade Física” OR “Pessoa com Limitação Física” OR “Pessoa com Necessidade Especial” OR “Pessoas com Deficiência Física” OR “Pessoas com Deficiências” OR “Pessoas com Deficiências Físicas” OR “Pessoas com Desvantagens” OR “Pessoas com Incapacidade” OR “Pessoas com Incapacidade Física” OR “Pessoas com Incapacidades” OR “Pessoas com Limitação Física” OR “Pessoas com Limitações Físicas” OR “Pessoas com Necessidade Especial” OR “Pessoas com Necessidades Especiais” OR “Disabled Persons” OR Handicapped OR “People with Disabilities” OR “Persons with Disabilities” OR “Physically Challenged” OR “Physically Disabled” OR “Physically Handicapped” OR “Deficiencia Física” OR “Deficiencias Físicas” OR “Persona con Deficiencia” OR “Persona con Deficiencia Física” OR “Persona con Desventaja” OR “Persona con Discapacidad” OR “Persona con Discapacidad Física” OR “Persona con Limitación Física” OR “Persona con Necesidad Especial” OR “Personas con Deficiencia” OR “Personas con Deficiencia Física” OR “Personas con Deficiencias” OR “Personas con Desventajas” OR “Personas con Discapacidad Física” OR “Personas con Discapacidades” OR “Personas con Discapacidades Físicas” OR “Personas con Limitación Física” OR “Personas con Limitaciones Físicas” OR “Personas con Necesidad Especial” OR “Personas con Necesidades Especiales” OR “Incapacidade funcional OR “Incapacidades funcionais” OR “Functional disability” OR “Functional disabilities”)*
*SCOPUS*	*(Leprosy OR “Hansen Disease” OR “Mycobacterium leprae”) AND (“Disabled Persons” OR “Disabled Person” OR “Functional disability” OR “Functional disabilities”)*
*Web Of Science*	*“Hansen Disease” OR “Hansen’s Disease” AND “Mycobacterium leprae” AND “Physically Challenged” AND “Physically Disabled” AND “Physically Handicapped” AND “Functional disabilities”*
*INFOLEP*	*“Leprosy” AND “Disabled Persons” AND “People with Disabilities”*
*Google Scholar*	*“Leprosy” AND “Disabled Persons” OR “People with Disabilities”*

### Selection of Studies and Mapping of Information

The obtained results were transferred to the web and mobile app for systematic reviews, Rayyan^(13)^. Duplicate citations were removed based on the outcomes in Rayyan, and the study selection was independently performed by two reviewers, examining titles and abstracts. Any disparities were resolved through discussion between the two reviewers, and if necessary, a third reviewer was involved. For documents meeting the inclusion criteria, a comprehensive reading was conducted to gather information on the impacts of physical disability caused by leprosy on quality of life. Exclusions were made for editorials, abstracts in event proceedings, research protocols, and documents not addressing leprosy in relation to quality of life and physical disability.

The Preferred Reporting Items for Systematic Review and Meta-Analysis (PRISMA)^(14)^ extension for scoping reviews Preferred Reporting Items for Systematic Reviews and Meta-Analyses extension for Scoping Reviews (PRISMA-ScR) was used to systematize the document inclusion process in the analysis^(15)^.

### Grouping, Summarizing, and Reporting of Results

Results and information were initially extracted by two reviewers and organized by year, authors, location, research design, objective, methodology, results, and conclusions, focusing on addressing the review question. Subsequently, for better visualization, a table was constructed based on the author, year, country, design, setting and research instrument. The studies were approached descriptively, and similar information was grouped to facilitate result relationships. To summarize the essential elements of the studies, Bardin’s content analysis^(16)^ was applied, a technique allowing the construction of thematic categories guided by the research question.

## RESULTS

**Figure 1:**
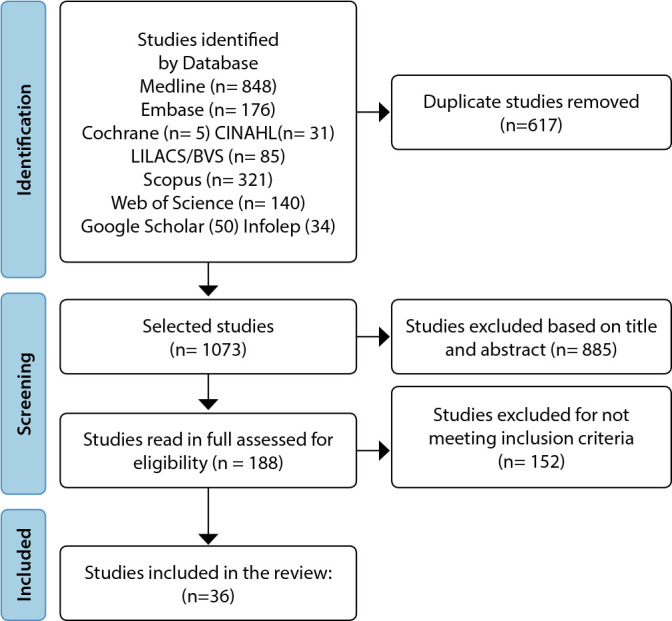
Inclusion and Exclusion Process of Identified Documents According to the Preferred Reporting Items for Systematic Review and Meta-Analysis (PRISMA)^(14)^

From the implemented search strategies, 1690 documents were identified. The MEDLINE/PubMed database had the highest number of works, followed by Scopus and Embase. Among these documents, 617 duplicates detected by the bibliographic management tool Rayyan^(13)^ were excluded. Out of the 1073 selected documents, 188 met the eligibility criteria and were fully evaluated. Of these, 36 articles composed the final sample, which were read in full and analyzed by two independent reviewers (Figure 1).

The articles that met the inclusion criteria were predominantly conducted in Brazil (19), followed by India (6), Ethiopia (2), Indonesia (3), Nigeria (2), and Nepal, Bangladesh, Colombia, and Malawi, each with one study. Regarding the study designs, the majority were quantitative cross-sectional studies (23), followed by descriptive studies (6), reviews (3), exploratory-qualitative study (1), quantitative-qualitative study (1), ecological study (1), and prospective cohort study (1).

In terms of language, 26 were in English, 8 in Portuguese, and 2 in Spanish. Through the content analysis stages of the included articles, the impacts of physical disability caused by leprosy on the quality of life of individuals were identified and synthesized into six thematic categories (Chart 2).

**Chart 2:** Thematic Categories from Data Analysis

Thematic Categories	Description
1) Impact on Activities of Daily Living	Instrumental activities include managing finances, dealing with transportation, shopping, meal preparation, using the phone and other communication devices, managing medications, and household maintenance; Basic activities encompass feeding oneself, going to the bathroom, choosing one’s clothes, dressing, personal grooming, maintaining continence, dressing, and bathing^(5,17-32)^.
2) Social Impact	There is a high prevalence of restricted social participation, social distancing, social exclusion leading to the loss of family bonds, and disruptions in social relationships^(19-21,24,26-37)^; difficulties related to education^(38)^.
3) Economic Impact	Low income leads to continued residence in nursing homes^(39)^; impediments/difficulties for employment^(20,32,37)^; government assistance^(26)^.
4) Psychological Impact	Stigma^(21,33,35,37)^, low mental well-being, and a high level of depression^(5,35,38,40)^; psychological stress^(41-42)^; fear, anxiety, sadness, guilt, shame, denial^(8-9,38)^; decreased prospects for marriage^(37,43)^, low self-esteem^(8)^; withdrawal, impulsivity, feelings of insecurity, dissatisfaction, and organicity^(39)^; sexual concerns^(43)^; self-control, avoidance, and escape strategies to cope with limitations^(22)^.
5) Impact of Pain	Pain is associated with the degree of physical disability and the multibacillary form, as a consequence of damage to peripheral nerves^(15,32,36,41)^, considered a late complication that generally impacts all domains of quality of life^(44)^.
6)Impact on Overall Well-being	Encompasses dimensions of life aspects: functional capacity; limitation due to physical aspect; pain; general health status; vitality; social aspects; emotional and mental health. These lead to functional disabilities, decreased work activity, and restriction of social life, causing damage and negative impacts on the quality of life^(7,10,23,40-41,45-47)^.

Various instruments were employed in the studies to assess physical disability and quality of life, with the most commonly used being: Screening of Activity Limitation and Safety Awareness (SALSA)^(18-19,21,24,29)^ World Health Organization Quality of Life - bref (WHOQOL-bref)^(10,30,36,42,46)^; Medical Outcomes Study 36 - Item Short - Form Health Survey (SF-36)^(5,31,36,46)^; Simplified Neurological Assessment^(19,26-27,36,39,41)^; Form for Assessment of the Degree of Disability^(28-29,32,40,45)^; Social Participation Scale^(18-19,24,27,29,33)^; Neuropathy-Specific Quality of Life Questionnaire (NeuroQol)^(20)^.

Regarding the study settings, specialized healthcare predominated in 14 studies^(5,8,20,23-24,26,28,31-34,36,43-44)^, followed by primary healthcare in 13 studies^(5,18,20,24,26,29,31,33,35,37,41,43,47)^, and hospital healthcare in 7 studies^(9,18-19,25,40,44,46)^.

In the Chart 3, detailed data on the 36 included studies are provided, categorized by year, author, country, setting and research instrument.

**Chart 3:** Included Studies by Second Authorship, Year, Country, Setting and Research Instrument.

Author/Year	Country	Design	Setting	Research Instrument
Ibikunle PO, Nwokeji SC (2017)^(33)^	Nigeria	Cross-sectional	Primary Care/Specialized Care	The Explanatory model interview catalogue (EMIC-a); The internalised stigma of mental illness scale (ISMI); The Participation scale: P-Scale;Eye, Hand & Foot impairment (EHF) Score.
Silva AC, Ferreira RC, Ferreira MA, Ribeiro MT(2014)^(17)^	Brazil	Cross-sectional	Hospital Care	The basic activities of daily living (BADL) e instrumental activities of daily living (IADL).
Abdela SG, Van Henten S, Abegaz SH, Bayuh FB, Zewdu FT, BerheFT, et al (2020)^(18)^	Ethiopia	Cross-sectional	Hospital Care	Screening of Activity Limitation and Safety Awareness (SALSA) scale e participation scale
Nascimento DDS, Ramos AN Jr, Araújo OD, Macêdo SF, Silva GVD, Lopes WMPS, et al(2020)^(19)^	Brazil	Cross-sectional	Primary Care	Screening of Activity Limitation and Safety Awareness (SALSA) scale e participation scale.
Achdiat PA, Ariyanto EF, Simanjuntak MN (2021)^(8)^	Indonesia	Review	^*^	^*^
Sousa NP, Silva MIB, Lobo CG, Barboza MCC, Abdon APV (2011)^(5)^	Brazil	Cross-sectional	Specialized Care	Medical Outcome Study 36-item Short-form Health Survey (SF36); a questionnaire addressing epidemiological, socioeconomic, and disease characteristics.
Carvalho MAJ, Lopes NTB, Santos TS, Santos KS, Farnocchi PG, Tavares CM (2013)^(39)^	Brazil	Descriptive	Primary Care	Sociodemographic Questionnaire; Simplified Neurological Evaluation Form.
Benedicto CB, Marques T, Milano AP, Galan NGA, Nardi ST, Duerksen Frank, et al (2017)^(20)^	Brazil	Descriptive	Specialized Care	Degree of Disability Assessment Form; Sociodemographic and Clinical Questionnaire; Human Figure Drawing (DFH); NeuroQol - Neuropathy-Specific Quality of Life Questionnaire (Brazilian Portuguese Version).
Govindasamy K, Jacob I, Solomon RM, Darlong J(2021)^(38)^	India	Cross-sectional	Primary Care	Standardized Questionnaire for Depression-(PHQ-9); Standardized Questionnaire for Anxiety Disorder-(GAD-7); Degree of Disability; and Socioeconomic Profile.
Ekeke N, Chukwu J, Nwafor C, Ogbudebe C, Oshi D, Meka A, Madichie N (2014)^(34)^	Nigeria	Descriptive-retrospective, cross-sectional	Specialized Care	Questionnaire-based interview; A retrospective descriptive desk analysis of leprosy case notification data for children 0 to 14 years in the 14 states in Southern Nigeria
Seshadri D, Khaitan BK, Khanna N, Sagar R (2015)^(43)^	India	Cross-sectional	Hospital Care	Clinical and Demographic Data; 52-item Anandaraj Dehabilitation Scale.
Van Dorst MMAR, Van Netten WJ, Waltz MM, Pandey BD, Choudhary R, Van Brakel WH (2020)^(35)^	Nepal	Cross-sectional	Primary Care	Sociodemographic Questionnaire; Patient Health Questionnaire (PHQ-9); Warwick-Edinburgh Mental Wellbeing Scale (WEMWBS); 5-Question Stigma Indicator-Affected Persons (5-QSI-AP).
Van Brakel WH, Sihombing B, Djarir H, Beise K, Kusumawardhani L, Yulihane R, et al (2020)^(21)^	Indonesia	Cross-sectional	Primary Care	Screening of Activity Limitation and Safety Awareness (SALSA) scale, Participation Scale, Jacoby Stigma Scale (anticipated stigma), Explanatory Model Interview Catalogue (EMIC) stigma scale And Discrimination assessment.
Beltrame RT, Marciano LHSC, Fonseca MDS; Prado RBR (2015)^(22)^	Brazil	Descriptive	Specialized Care	Sociodemographic Questionnaires, Clinical aspects of the disease, and Functional Independence in performing ADLs and IADLs; Checklist related to the type of visible physical disability found in the patient; and the Folkman and Lazarus Coping Strategies Inventory, adapted to Portuguese.
Viana LDS, Aguiar MIFD, Vasconcelos PFD, Aquino DMCD (2017)^(23)^	Brazil	Descriptive, with quantitative approach	Specialized Care	Physical Domain of the World Health Organization Quality of Life (WHOQOL-BREF) and the “Sensory Abilities” and “Autonomy” facets of WHOQOL-OLD.
Van Veen NHJ, Hemo DA, Bowers RL, Pahan D, Negrini JF, Velema JP, et al (2011)^(24)^	Bangladesh	Prospective Cohort	Hospital Care	Screening of Activity Limitation and Safety Awareness (SALSA) scale, Participation Scale
Hurtado MN, Atehortua MC, Bravo, J (2003)^(25)^	Colombia	Descriptive	Primary Care	International Classification of Functioning; Sociodemographic Form.
Sharma M, Saxena V (2019)^(26)^	India	Cross-sectional, observational	Specialized Care	Demographic Socioeconomic and Epidemiological Data; Availability of various government and non-government facilities; Body Mass Index - BMI; WHO Disability Grading.
Vieira CSCA, Lobato ML, Figueira MCS, Amaral MCE, Vilela MFG, Silva EM (2018)^(27)^	Brazil	Ecological, analytical	Primary Care	Simplified Neurological Evaluation; Sociodemographic and Clinical Profile; Screening of Activity Limitation and Safety (SALSA); Participation Scale.
Nardi SMT, Paschoal VD, Zanetta DMT (2012)^(28)^	Brazil	Cross-sectional	Specialized Care	Screening of Activity Limitation and Safety Awareness (SALSA) scale
Monteiro LD, Alencar CH, Barbosa JC, Novaes CCBS, Silva RCP, HeukelbachI J (2014)^(29)^	Brazil	Cross-sectional	Primary Care	Simplified Neurological Evaluation; Screening of Activity Limitation and Safety Awareness (SALSA); Participation Scale.
D’Azevedo SSP, et al (2021)^(30)^	Brazil	Cross-sectional	Primary Care	Sociodemographic and Clinical Questionnaire and the World Health Organization Disability Assessment Schedule (WHODAS).
Reis FJJ, Lopes D, Gosling AD, Gomes MK (2014)^(41)^	Brazil	Cross-sectional	Hospital Care	Douleur neuropathique en 4 questions questionnaire; 12-item General Health Questionnaire; World Health Organization quality of life (WHOQOL-BREF)
Govindharaj P, Srinivasan S, Darlong J (2018)^(9)^	India	Cross-sectional descriptive	Hospital Care	Semi-structured Questionnaire.
Ramos JM, Alonso-Castaneda B, Eshetu D, Lemma D, Reyes F, Belinchon I, et al (2014)^(44)^	Ethiopia	Cross-sectional	Specialized Care	Neuropathic Pain Symptom Inventory (NPSI) questionnaire
Rodini FCB, Gonçalves M, Barros ARSB, Mazzer N, Elui LMC, Fonseca MCR (2010)^(36)^	Brazil	Cross-sectional	Specialized Care	Medical Outcome Study 36-item Short-form health Survey (SF36)
PatilA, Mayur SS (2021)^(42)^	India	Cross-sectional	Hospital Care	World Health Organization quality of life (WHOQOL-BREF); Questionnaire and mental health status by the SelfReporting questionnaire
Gaudenci EM, Nardelli GG, Almeida Neto OP, Malaquias BSS Carvalho BT, Pedrosa LAK (2015)^(40)^	Brazil	Cross-sectional, quantitative, descriptive, and analytical	Specialized Care	Clinical and Socioeconomic Questionnaire; Beck Depression Inventory- BDI for Depression Assessment; World Health Organization Quality of Life (WHOQOL-BREF).
Costa RMPC, Mendes LCB(2020)^(10)^	Brazil	Integrative Review	^*^	^*^
Govindharaj P, Srinivasan S, Darlong J(2018)^(45)^	India	Cross-sectional	Hospital Care	World Health Organization quality of life (WHOQOL-BREF)
Lustosa AA, Nogueira LT, Pedrosa JIS, Teles JBM, Campelo V (2011)^(46)^	Brazil	Cross-sectional	Specialized Care	Medical Outcome Study 36-item Short-form Health Survey (SF36), Clinical, Sociodemographic, and Epidemiological Data.
Chingu D, Duncan M, Amosun S (2013)^(47)^	Malawi	Cross-sectional	Primary Care	World Health Organization quality of life (WHOQOL-BREF)
Schuller I, Van Brakel WH, Van Vliet I, Beise K, Wardhani L, Silwana S, et al (2010)^(37)^	Indonesia	Quantitative-qualitative	Primary Care	Interviews and Focus Groups.
Prado GD, Prado RBR, Marciano LHSC, Nardi SMT, Cordeiro JA, Monteiro HL (2011)^(31)^	Brazil	Cross-sectional	Specialized Care	World Health Organization degree of physical disability classification (WHO-DG), the International Physical ActivityQuestionnaire (IPAQ) and the Medical Outcome Study 36-item Short-form health Survey (SF36)
Gonçalves M, Prado MAR, Silva SS, Santos KS, Araujo PN, Fortuna CM (2018)^(32)^	Brazil	Qualitative	Specialized Care	Semi-structured Interviews.
Barcelos RMFM, Sousa GS, Almeida MV, Palacio FGL, Gaíva MAM, Ferreira SMB^(7)^	Brazil	Review	^*^	^*^

* *As this is a literature review, there is no specific setting, and the instrument is the methodology itself.*

## DISCUSSION

The studies encompassed in this review enabled the identification of the impacts of physical disability resulting from leprosy on the quality of life of individuals treated within the Health Care Network. Brazil^(5,7,10,17-23,27-32,36,40-41,46)^ emerged as the leading country in publication, underscoring its epidemiological significance, predominantly through cross-sectional studies. The studies included in this review revealed the following impacts instigated by the disease:

- Impact on activities of daily living^(5,17-18,20-32)^;
- Socio-economic impact^(19-21,24,26-37,39)^;
- Psychological impact^(5,8,22,35,38-43)^;
- Impact of pain^(15,32,36,41,44)^;
- Impact on overall well-being^(7,10,23,40-41,45-47)^.

When assessing the impact of limitations in activities of daily living, losses in the physical aspect were observed, resulting in diminished levels of quality of life in this population. Physical limitations encompass pain, reduced mobility, deformities negatively impacting activities of daily living such as self-care, mobility, feeding, personal hygiene, dressing, undressing, and footwear. Moreover, other daily activities such as managing one’s house, shopping, and using transportation are affected, disrupting social integration^(17-18,21-22,28-29)^.

The studies depicted a robust relationship between GIF 1 and 2, the multibacillary form, and the degree of dependence on Basic Activities of Daily Living (BADL) and Instrumental Activities of Daily Living (IADL)^(15-17,21-24,26-28,30-31)^. A high prevalence of physical disability was observed, including amputations and visible deformities in the elderly population^(15)^. It is noteworthy that even after being cured of the disease, there are cases where the impacts are permanent^(26)^. On the other hand, reconstructive surgery was found to be effective in improving activities of daily living. Therefore, it is a strategy to enhance the quality of life for these individuals^(23)^.

The specific instrument for evaluating the limitation of activities of daily living, SALSA, was one of the most used^(17-19,21,24,27-29)^. In this sense, this scale can support services in treatment since GIF2 at diagnosis indicates late detection and intervening factors such as operational difficulties in services, lack of information about signs and symptoms, difficulty accessing services, and qualified professionals^(29)^.

Addressing the impact of social and economic aspects revealed that the majority of the affected population is male, aged 40 and above, with low education, married or widowed, unemployed or without occupation, and low family income^(21,26,39,44-45)^. Therefore, it indicates the relationship of GIF linked to social and economic aspects, leading to low scores in quality of life^(44)^. While leprosy continues to affect people more vulnerable to socioeconomic determinants, health professionals’ interventions related to health education and self-care guidance can positively impact the quality of life of those with the disease^(9)^.

In this context, a study with 26 people affected by leprosy assessed the quality of life of this group before and after one year of implementing a self-care guide. The authors identified improvements in pain, motor function, skin conditions, and social aspects^(36)^. Regarding social aspects, the restriction of social participation has been associated with limitations in learning and applying knowledge, communication, mobility, self-care, interpersonal relationships, and community life. The studies included in the review that applied this scale found high levels of restriction of social participation^(18-19,24,27-28,33)^.

When portraying the impacts of psychological aspects, the stigma and self-stigma caused by the disease generate negative social and emotional consequences that can result in high levels of social distancing in the community^(8)^. These cases are more frequent in individuals with GIF2, lower family income, and lower educational levels. Regarding gender, women suffer from stigmatization to a greater extent due to restrictions on social participation and lower prospects of marriage^(21,33,37,40)^. The idealized aesthetic standard is often more intense for women, establishing a higher level of disease-related stigma^(20,32)^.

To address stigma, understanding the experience of patients affected by leprosy can enable the planning of health service strategies. For this purpose, the Stigma Scale for People Affected by Leprosy (EMIC-AP) and the Participation Scale can be used, as recommended in the Clinical Protocol and Therapeutic Guidelines for Leprosy (PCDT leprosy)^(48)^. Additionally, these scales can contribute to improving the quality of care, promoting comprehensive care, and guiding actions in psychosocial care and mental health^(48)^.

Low levels of psychological well-being and high levels of depression are more common in people with physical disability due to leprosy compared to the general population. This is due to the significant stigma of the disease that intensifies feelings such as anxiety, sadness, fear, low self-esteem, sleep disorders, and even suicidal thoughts. Consequently, many affected individuals feel guilt and shame for contracting the disease, leading to impairments in interpersonal relationships^(8,20,29,34)^. Psychotherapy can facilitate understanding of the physical and emotional condition, as well as the reorganization of body image and improvement of self-esteem^(5)^.

Strategies such as health education and guidance manuals contribute to increased education levels, clarification on the topic, and access to appropriate multidisciplinary treatment^(36,49)^. It is understood that multidisciplinary intervention programs for health education involve interventions for holistic care, physical, mental, and psychosocial health for the community and families of patients, consequently capable of improving quality of life indices^(36)^.

In this context, the continuous and humanized evaluation of disease progression and active case finding are indispensable tools in preventing complications, as well as improving psychosocial well-being^(35)^. In the impact of pain, neuropathic pain stands out, recognized as another late complication in leprosy patients.

There is a high prevalence of reported pain, leading to significant changes in sleep and daily activities^(42)^, associated with the degree of physical disability, social participation^(27-28)^, and psychological distress^(36)^. Progressive damage to peripheral nerves can lead to a chronic state of pain, even after the end of treatment^(41)^. Chronic pain is associated with psychological distress and is a significant predictor of poor quality of life. Therefore, pain management can contribute to reducing psychological disorders and improving quality of life^(41)^.

In the evaluation of quality of life, the domains that presented lower scores were limitation due to physical aspects, pain, and emotional aspects^(17)^. It was found that the greatest impairment was related to the loss/reduction of sensitivity^(5)^ with a predominance of disabling forms of leprosy^(21)^. Physical disabilities due to leprosy are often not reversible; however, it is possible to achieve quality of life when prevention actions are established.

In this regard, a study^(36)^ used a manual for the prevention of disabilities, resulting in an improvement in the quality of life in the pain and social aspects domains of the SF-36 questionnaire. Additionally, they found that pain, discomfort, and dependence on medication were related to greater implications in the compromised physical domain^(36)^. Other studies indicate that the physical domain was the most impacted, with an inversely proportional relationship to GIF, i.e., the higher the GIF, the lower the quality of life scores^(36,44)^. As a result, a decrease in work activities and restriction of social life was observed, leading to impairments in quality of life^(17,25-26,40,47)^.

The term Health-Related Quality of Life (HRQoL) is more specific and involves the impact of a disease on the quality of life^(10)^. It was found that the higher the degree of disease progression and the establishment of disabling forms, the worse the HRQoL. To analyze the quality of life of leprosy patients, five determinants of HRQoL were identified: late diagnosis, multibacillary forms, reactions, GIF2 at diagnosis, and prejudices^(10)^.

In this review, there was a predominance of studies conducted in specialized care, and consequently, the impacts of physical disabilities were more significant. In the evaluation of quality of life and activity limitations, the application of instruments such as WHOQOL-bref^(10,30,36,42,46)^, SF-36^(5,31,36,46)^, and SALSA^(18-19,21,24,29)^ was identified in Primary Care, Specialized Care, and Hospital Care, as per the supplementary document.

It is worth highlighting the representation of nursing in the Health Care Network in Brazil. The nurse, as the leader of the nursing team, is responsible for planning and providing information about leprosy, prevention of disabilities, self-care, treatment, as well as conducting active case-finding activities. Thus, nursing consultations become essential in establishing a connection between the nurse and the person with leprosy^(50)^.

Finally, the training of healthcare professionals, especially in nursing, health education activities, defining professional roles, and using specific instruments in nursing care can contribute to effective care and an improvement in the quality of life of patients. It is suggested that scales such as WHOQOL-bref, SF-36, and SALSA, as well as those indicated in the Clinical Protocol and Therapeutic Guidelines for Leprosy (EMIC-AP and Participation Scale)^(48)^, be applied during nursing consultations, as they can support nursing care actions.

### Study limiations

We recognize several inherent limitations in this review. Firstly, while our selection of databases was comprehensive, it may have overlooked potential contributions from other sources. Additionally, the predominance of studies conducted in Brazil could restrict the applicability of the results to different contexts, underscoring the necessity for further research in other countries. The diversity in methods and assessment instruments across the included studies might introduce variations in the results, making a more consistent synthesis of evidence challenging. Lastly, the absence of a detailed analysis of the methodological quality of the included studies could impact the reliability of the presented results. These considerations offer a critical perspective on the conclusions drawn from this review, underscoring the importance of future research to address these limitations and enhance our understanding of the impacts of leprosy on quality of life.

### Contributions of the Study to the Nursing Field:

The findings from this work play a crucial role in mitigating the impacts of leprosy on individuals’ quality of life, providing tangible benefits for healthcare assistance in the areas of prevention, promotion, and rehabilitation. By offering a comprehensive reflection on the various aspects involved in holistic individual care, the study promotes the coordination of discussions among various stakeholders engaged in health policy planning. The goal is to enhance leprosy control actions and improve overall health outcomes.

## CONCLUSIONS

Our study facilitated the identification and synthesis of the impacts of physical disability caused by leprosy and its repercussions on the quality of life of individuals treated within the Health Care Network. The effects of leprosy on quality of life were observed in activities of daily living, social and economic engagement, mental health, overall well-being, and the experience of pain. The literature mapping underscored the breadth of the topic and emphasized the crucial need for disease control measures, such as early and timely diagnosis, which is pivotal in preventing the development of physical disability leading to various complications that directly affect quality of life.

As a result, actions focused on prevention, promotion, and rehabilitation are deemed essential, coupled with continuous education for healthcare professionals to enable effective case tracking and monitoring. Nurses, as integral members of the multidisciplinary team, play a critical role in caring for this population by providing disease clarification, preventing disabilities, promoting self-care, and facilitating treatment. They also actively contribute to the identification of new cases. It is noteworthy that, in addition to clinical knowledge, the utilization of measurement instruments can enhance assistance to this population.
