# Supplementary material for: Leprosy and its impact on the quality of life of people with physical disabilities: a scoping review
Source: Rev Bras Enferm. 2024 Jul 15;77(Suppl 3):e20230101. doi: 10.1590/0034-7167-2023-0101 (PMC11253837; doi:10.1590/0034-7167-2023-0101)
Supplement: Translation

# Translation: Hanseníase e impactos na qualidade de vida de pessoas com incapacidades físicas: revisão de escopo

**DOI:** 10.1590/0034-7167-2023-0101pt

**Authors:** Douglas Moreira de Araujo, Elisa Camila de Souza e Silva, Helen Vaz da Silva Gomes, Fábio da Costa Carbogim, Gesner Francisco Xavier, Angélica da Conceição Oliveira Coelho

**Affiliations:** I Universidade Federal de Juiz de Fora. Juiz de Fora, Minas Gerais, Brasil; II Universidade Federal de Minas Gerais. Belo Horizonte, Minas Gerais, Brasil

**Keywords:** Hanseníase, Mycobacterium Leprae, Pessoas com Incapacidade Física, Pessoa com Incapacidade, Qualidade de Vida

## Abstract

**Objetivos:**

identificar e sintetizar, a partir da literatura, os impactos da incapacidade física causada pela hanseníase na qualidade de vida dos indivíduos atendidos na Rede de Atenção à Saúde.

**Métodos:**

trata-se de uma revisão de escopo, realizada de acordo com as recomendações do JBI. As bases de dados utilizadas na busca foram *Medical Literature Analysis and Retrieval System Online, Cochrane library, Web of Science, Lilacs, Cumulative Index to Nursing and Allied Health Literature, Scopus, Embase, Leprosy Information Services* e *Google Scholar*.

**Resultados:**

foram identificados 1690 documentos, dos quais 36 compuseram a revisão. A incapacidade física causada pela hanseníase impacta na qualidade de vida no âmbito de atividades de vida diária, socioeconômico, psicológico, da dor e no bem-estar geral.

**Conclusões:**

foram identificados os comprometimentos causados pela incapacidade física na qualidade de vida, indicando a necessidade de ações de prevenção, promoção e reabilitação, como rastreamento, acompanhamento dos casos e educação em saúde.

## INTRODUÇÃO

A hanseníase é uma doença tropical, negligenciada, causada pelo *Mycobacterium leprae*, que afeta principalmente a pele e os nervos periféricos, com risco de progressão para incapacidades físicas (IF)^(1)^. O manejo inadequado da hanseníase, bem como diagnóstico e tratamentos tardios, contribuem para o pior desfecho clínico^(2)^. Neste contexto, destaca-se a importância de os profissionais de saúde estarem preparados para atuar junto a este agravo, em especial a enfermagem, que representa o maior número de profissionais na Rede de Assistência à Saúde^(3)^.

Em 2021, foram registrados mais de 140 mil novos casos no mundo, com a Índia em primeiro lugar em número absoluto de casos, seguido do Brasil. Quanto ao grau de incapacidade física, foram mais de 7 mil casos diagnosticados com grau de incapacidade física 2 (GIF2)^(1)^. No Brasil, em 2022, 1.449 pessoas foram diagnosticadas com GIF 2 e 4.041 com GIF 1, correspondendo respectivamente a 11,6% e 32,4% dos casos. Cabe salientar que os estados brasileiros de Mato Grosso e Tocantins são os que apresentam maiores taxas de detecção de novos casos da doença^(4)^. A detecção precoce interrompe a progressão da hanseníase, evitando o comprometimento físico e, por consequência, prejuízos sociais, emocionais, psíquicos e piores níveis de qualidade de vida^(5)^. Segundo a Organização Mundial da Saúde (OMS), qualidade de vida é “a percepção do indivíduo de sua inserção na vida, no contexto da cultura e sistemas de valores nos quais ele vive e em relação aos seus objetivos, expectativas, padrões e preocupações”^(6)^.

Na área de saúde, a avaliação da qualidade de vida geralmente é captada por instrumentos com propriedades psicométricas adequadas que permitem detectar alterações físicas, emocionais e sociais^(5-6)^. Na literatura sobre hanseníase, revisão identificou que os instrumentos mais frequentemente utilizados para avaliar qualidade de vida são o *WHOQOL-bref* e o *Medical Outcomes Study 36 - Item Short - Form Health Survey (SF-36)* ^(7)^. Este mesmo estudo destacou que o pior nível de qualidade de vida está associado ao comprometimento físico, às reações hansênicas, às incapacidades físicas, à dor neuropática e ao estigma^(7)^.

Por outro lado, o aumento da qualidade da assistência para essa população pode implicar em ações de autocuidado, na diminuição das sequelas, dos impactos emocionais e sociais e no envolvimento para o tratamento e a reabilitação^(8-9)^. Nesse sentido, detectar convergência entre as sequelas da doença e aspectos de qualidade de vida pode contribuir com o direcionamento das ações de saúde. Apesar do conhecimento científico sobre os danos físicos e sociais acerca da hanseníase serem amplamente estudados, ainda são escassos os mapeamentos de evidências científicas sobre incapacidades físicas e impactos na qualidade de vida dessa população^(7-10)^.

Portanto, estudos de revisão sobre a qualidade de vida dos pacientes de hanseníase com incapacidade física se justificam pela necessidade de contribuir para políticas focadas na prevenção e promoção da saúde, de forma a reduzir os prejuízos causados pela doença, além de possibilitar que os profissionais de saúde busquem alternativas de tratamentos e intervenções efetivas para cada caso.

## OBJETIVOS

Identificar e sintetizar, a partir da literatura, os impactos da incapacidade física causada pela hanseníase na qualidade de vida dos indivíduos atendidos na Rede de Atenção à Saúde.

## MÉTODOS

### Desenho do Estudo

Trata-se de uma revisão de escopo fundamentada na metodologia do *The Joanna Briggs Institute* (JBI) que busca contemplar toda a literatura científica, com vista a obter resultados amplos e abrangentes^(11)^. Para conduzir o estudo, seguiram-se os cinco passos metodológicos: 1) identificação da questão norteadora; 2) identificação de estudos relevantes; 3) seleção de estudos; 4) mapeamento das informações; 5) agrupamento, resumo e relato dos resultados^(12)^. Além disso, cabe destacar que para esta revisão foi adotado o conceito de qualidade de vida da Organização Mundial da Saúde^(6)^.

### Identificação da questão norteadora

A questão norteadora da revisão foi elaborada de acordo com o acrônimo PCC^(9)^ (População, Conceito e Contexto): “Qual o impacto da incapacidade física na qualidade de vida das pessoas afetadas pela hanseníase atendidas na Rede de Atenção à Saúde?”. Para tanto, denominou-se o P (população) como pessoas acometidas pela hanseníase, o C (conceito) como impacto da incapacidade física na qualidade de vida e o C (contexto) como todos os níveis de atendimento à saúde. O protocolo da presente revisão foi registrado na plataforma Open Science Framework, DOI 10.17605/OSF.IO/ZB4WR.

### Identificação de estudos relevantes

Inicialmente foi realizada uma busca na base de dados *Medical Literature Analysis and Retrieval System Online* (MEDLINE) via PubMed, para identificar estudos que analisaram o impacto na qualidade de vida de pacientes que desenvolveram incapacidade física devido à Hanseníase. Para encontrá-los, foram utilizados os descritores: *“Leprosy”, “Hansen Disease”, “Disabled Persons”, “People with Disabilities”* e seus possíveis sinônimos, de acordo com a plataforma DeCS/MeSH e os operadores booleanos “*AND*” e “*OR*”. Vale ressaltar que não foram utilizados os descritores “Níveis de Atenção à Saúde” e “Qualidade de vida” por limitarem os resultados da busca.

A estratégia de busca foi adaptada de acordo com a especificidade de cada base de dados (Quadro 1): MEDLINE (PubMed), *Cochrane Library, Web of Science*, Literatura Latino-americana e do Caribe em Ciências da Saúde (Lilacs) via Biblioteca Virtual de Saúde (BVS), *Cumulative Index to Nursing and Allied Health Literature* (CINAHL), Scopus e Embase. Fontes de literatura cinzenta foram pesquisadas a *Leprosy Information Services* (INFOLEP) e o *Google Scholar*, em suas primeiras cinco páginas. Consideraram-se estudos publicados em inglês, português e espanhol publicados até 30 de agosto de 2023.

**Quadro 1:** Estratégia de busca de acordo com a base de dados

Base de dados	Estratégia de busca
*Medline via Pubmed*	((“Leprosy”[Mesh] OR (Hansen’s Disease) OR (Hansen Disease) OR (Hansen^*^) OR (Lepr^*^)) OR (“Mycobacterium leprae”[Mesh] OR (Mycobacterium leprae))) AND (“Disabled Persons”[Mesh] OR (Disabled Person) OR (Person, Disabled) OR (Persons, Disabled) OR (Handicapped) OR (People with Disabilities) OR (Disabilities, People with) OR (People with Disability) OR (Persons with Disabilities) OR (Disabilities, Persons with) OR (Disability, Persons with) OR (Persons with Disability) OR (Physically Handicapped) OR (Handicapped, Physically) OR (Physically Disabled) OR (Disabled, Physically) OR (Physically Challenged))
*Cochrane*	*(Leprosy OR “Hansen Disease” OR “Mycobacterium leprae”) AND (“Disabled Persons” OR “Disabled Person” OR “Functional disability” OR “Functional disabilities”)*
*EMBASE*	*(‘leprosy’/exp OR leprosy OR ‘mycobacterium leprae’/exp OR ‘mycobacterium leprae’) AND (‘disabled persons’/exp OR ‘disabled persons’ OR ((‘disabled’/exp OR disabled) AND (‘persons’/exp OR persons)) OR ‘disabled person’/exp OR ‘disabled person’ OR ‘functional disability’/exp OR ‘functional disability’ OR ‘functional disabilities’)*
*CINAHL*	*(Leprosy OR “Hansen Disease” OR “Mycobacterium leprae”) AND (“Disabled Persons” OR “Disabled Person” OR “Functional disability” OR “Functional disabilities”)*
Lilacs via BVS	*(TW: Hanseníase OR “Doença de Hansen” OR Lepra OR “Hansen Disease” OR “Hansen’s Disease” OR “Enfermedad de Hansen” OR “Mal de Hansen” OR “Mycobacterium leprae” OR “Bacilo da Hanseníase” OR “Bacilo de Hansen” OR “Mycobacterium leprae” OR “Bacilo de Hansen” OR “Bacilo de la Hanseniasis” OR “Bacilo de la Lepra”) AND (TW: “Deficiência Física” OR “Deficiências Físicas” OR “Deficiente Físico” OR “Limitação Física” OR “Pessoa com Deficiência Fisica” OR “Pessoa com Desvantagem” OR “Pessoa com Incapacidade” OR “Pessoa com Incapacidade Física” OR “Pessoa com Limitação Física” OR “Pessoa com Necessidade Especial” OR “Pessoas com Deficiência Física” OR “Pessoas com Deficiências” OR “Pessoas com Deficiências Físicas” OR “Pessoas com Desvantagens” OR “Pessoas com Incapacidade” OR “Pessoas com Incapacidade Física” OR “Pessoas com Incapacidades” OR “Pessoas com Limitação Física” OR “Pessoas com Limitações Físicas” OR “Pessoas com Necessidade Especial” OR “Pessoas com Necessidades Especiais” OR “Disabled Persons” OR Handicapped OR “People with Disabilities” OR “Persons with Disabilities” OR “Physically Challenged” OR “Physically Disabled” OR “Physically Handicapped” OR “Deficiencia Física” OR “Deficiencias Físicas” OR “Persona con Deficiencia” OR “Persona con Deficiencia Física” OR “Persona con Desventaja” OR “Persona con Discapacidad” OR “Persona con Discapacidad Física” OR “Persona con Limitación Física” OR “Persona con Necesidad Especial” OR “Personas con Deficiencia” OR “Personas con Deficiencia Física” OR “Personas con Deficiencias” OR “Personas con Desventajas” OR “Personas con Discapacidad Física” OR “Personas con Discapacidades” OR “Personas con Discapacidades Físicas” OR “Personas con Limitación Física” OR “Personas con Limitaciones Físicas” OR “Personas con Necesidad Especial” OR “Personas con Necesidades Especiales” OR “Incapacidade funcional OR “Incapacidades funcionais” OR “Functional disability” OR “Functional disabilities”)*
*SCOPUS*	*(Leprosy OR “Hansen Disease” OR “Mycobacterium leprae”) AND (“Disabled Persons” OR “Disabled Person” OR “Functional disability” OR “Functional disabilities”)*
*Web Of Science*	*“Hansen Disease” OR “Hansen’s Disease” AND “Mycobacterium leprae” AND “Physically Challenged” AND “Physically Disabled” AND “Physically Handicapped” AND “Functional disabilities”*
*INFOLEP*	*“Leprosy” AND “Disabled Persons” AND “People with Disabilities”*
*Google Scholar*	*“Leprosy” AND “Disabled Persons” OR “People with Disabilities”*

### Seleção dos estudos e mapeamento das informações

Os resultados recuperados foram exportados para a plataforma *web and mobile app for systematic reviews* (*Rayyan*)^(13)^. A partir dos resultados obtidos no Rayyan, foram eliminadas citações duplicadas e a seleção dos estudos estabelecida por dois revisores independentes a partir da leitura dos títulos e resumos. As divergências foram solucionadas pela discussão entre os dois revisores e, quando necessário, a participação de um terceiro revisor. Para os documentos que atenderam aos critérios de inclusão, procedeu-se à leitura dos documentos na sua integralidade, buscando informações sobre os impactos da incapacidade física causada pela hanseníase na qualidade de vida. Foram excluídas cartas ao editor, resumos em anais de eventos, protocolos de pesquisa e documentos que não abordassem a hanseníase relacionada à qualidade de vida e à incapacidade física. A extensão do *Preferred Reporting Items for Systematic Review and Meta-Analysis* (PRISMA)^(14)^ para revisões de escopo *Preferred Reporting Items for Systematic Reviews and Meta-Analyses extension for Scoping Reviews* (PRISMA-ScR) foi utilizada para organizar o processo sistemático de inclusão de documentos na análise^(15)^.

### Agrupamento, resumo e relato dos resultados

Os resultados e as informações foram inicialmente extraídos por dois revisores e organizados de acordo com o ano, autores, local, delineamento da pesquisa, objetivo, metodologia, resultados e conclusões, além de focar na resposta à pergunta da revisão. Em seguida, para melhor visualização, foi construído um quadro com base no autor, ano, país, delineamento, cenário e instrumento de pesquisa. Os estudos foram abordados de forma descritiva, e as informações semelhantes foram agrupadas para facilitar a relação dos resultados. Para resumir os elementos essenciais dos estudos, utilizou-se a análise de conteúdo de Bardin^(16)^, técnica que possibilitou a construção de categorias temáticas, orientadas pela questão de pesquisa.

## RESULTADOS

A partir das estratégias de busca organizadas para a realização dessa pesquisa, foram identificados 1690 documentos. A base de dados com maior número de trabalhos foi a *MEDLINE/PubMed*, seguida da *Scopus* e *Embase*. Destes documentos, foram excluídos 617 duplicados detectados pela ferramenta de gestão bibliográfica *Rayyan* ^(13)^. De 1073 documentos selecionados e 188, por critérios de elegibilidade, foram avaliados na integra. Destes, compuseram a amostra final 36 artigos, que foram lidos na íntegra e analisados por dois revisores independentes (Figura 1).

**Figura 1:** Processo de inclusão e exclusão dos documentos encontrados de acordo com as recomendações do *Preferred Reporting Items for Systematic Review and Meta-Analys* PRISMA^(14)^

Os artigos que atenderam ao critério de inclusão foram em sua maioria realizados no Brasil (19), seguido da Índia (6), Etiópia (2), Indonésia (3), Nigéria (2) e Nepal, Bangladesh, Colômbia e Malawi com 01 estudo cada. Com relação ao delineamento dos estudos, em grande parte, foram estudos quantitativos transversais (23), seguidos dos descritivos (6), revisão (3), estudo exploratório-qualitativo (1), estudo quantitativo-qualitativo (1), estudo ecológico (1) e coorte prospectiva (1).

Quanto ao idioma, foram 26 na língua inglesa, 8 em português e 2 em espanhol. A partir das etapas da análise de conteúdo dos artigos incluídos, foram identificados os impactos da incapacidade física causada pela hanseníase na qualidade de vida dos indivíduos, sintetizados em seis categorias temáticas (Quadro 2).

**Quadro 2:** Categorias temáticas a partir da análise dos dados

Categoria	Descrição
1) Impacto nas atividades de vida diária	Atividades instrumentais de vida diáriagerenciar as finanças, lidar com transporte, fazer compras, preparar refeições, usar o telefone e outros aparelhos de comunicação, gerenciar medicações, manutenção das tarefas domésticas e da casa; Atividades básicas de vida diáriaalimentar-se, ir ao banheiro, escolher a própria roupa, arrumar-se e cuidar da higiene pessoal, manter-se continente, vestir-se, tomar banho^(5,17-32)^
2)Impacto social	Alta prevalência à restrição da participação social; distanciamento social; exclusão social com perda do vínculo familiar; transtornos nas relações sociais^(19-21,24,26-37)^; dificuldades relacionadas à educação^(38)^
3)Impacto econômico	Baixa renda, o que leva a continuar a residir em asilos^(39)^; impedimento/dificuldades para o trabalho^(20,32,37)^; auxílio governamental^(26)^
4)Impacto psicológico	Estigma^(21,33,35,37)^, baixo bem estar mental e alto nível de depressão^(5,35,38,40)^; estresse psicológico^(41-42)^; medo, ansiedade, tristeza, culpa, vergonha, negação^(8-9,38)^, diminuição de perspectivas para casamento^(37,43)^, baixo autoestima^(8)^; retraimento, impulsividade, sentimento de insegurança, descontentamento e organicidade^(39)^; preocupações sexuais^(43)^; estratégias de autocontrole, esquiva e fuga para enfrentar as limitações^(22)^.
5)Impacto da Dor	A dor está associada ao grau de incapacidade física e à forma multibacilar, como consequência dos danos nos nervos periféricos^(15,32,36,41)^, sendo considerada uma complicação tardia que impacta de modo geral em todos domínios da qualidade de vida^(44)^
6)Impacto no bem-estar geral	Inclui as dimensões dos aspectos de vida: capacidade funcional; limitação por aspecto físico; dor; estado geral de saúde; vitalidade; aspectos sociais; emocionais e a saúde mental. Essas levam a incapacidades funcionais, diminuição da atividade laboral e restrição da vida social o que causa prejuízos e impactos negativos na qualidade de vida^(7,10,23,40-41,45-47)^.

Dentre os diversos instrumentos utilizados nos estudos para avaliação da incapacidade física e qualidade de vida, os mais utilizados foram: *Screening of Activity Limitation and Safety Awareness (SALSA)* ^(18-19,21,24,29)^ *; World Health Organization Quality of Life - bref (WHOQOL-bref)* ^(10,30,36,42,46)^; *Medical Outcomes Study 36 - Item Short - Form Health Survey* (SF-36)^(5,31,36,46)^; Avaliação neurológica simplificada^(19,26-27,36,39,41)^; Formulário de Avaliação do Grau de Incapacidade^(28-29,32,40,45)^; Escala de participação social^(18-19,24,27,29,33)^; *Neuropathy-Specific Quality of Life Questionnaire* (NeuroQol)^(20)^.

Quanto aos cenários dos estudos, a atenção especializada à saúde prevaleceu em 14 estudos^(5,8,20,23-24,26,28,31-34,36,43-44)^, seguida da atenção básica à saúde, em 13 estudos^(5,18,20,24,26,29,31,33,35,37,41,43,47)^, e a atenção hospitalar à saúde, em 7 estudos^(9,18-19,25,40,44,46)^.

No Quadro 3, encontram-se detalhados dados dos 36 estudos incluídos de acordo com autor, ano, país, cenário e instrumento de pesquisa.

**Quadro 3:** Estudos incluídos segundo autoria, ano, país, cenário e instrumento de pesquisa.

Autor /Ano	País	Delineamento	Cenário	Instrumento de pesquisa
Ibikunle PO, Nwokeji SC (2017)^(33)^	Nigéria	Transversal	Atenção básica/atenção especializada	*The Explanatory model interview catalogue (EMIC-a); The internalised stigma of mental illness scale (ISMI); The Participation scale: P-Scale;Eye, Hand & Foot impairment (EHF) Score.*
Silva AC, Ferreira RC, Ferreira MA, Ribeiro MT(2014)^(17)^	Brasil	Transversal	Atenção hospitalar	*The basic activities of daily living (BADL) e instrumental activities of daily living (IADL).*
Abdela SG, Van Henten S, Abegaz SH, Bayuh FB, Zewdu FT, BerheFT, et al (2020)^(18)^	Etiópia	Transversal	Atenção hospitalar	*Screening of Activity Limitation and Safety Awareness (SALSA) scale e participation scale*
Nascimento DDS, Ramos AN Jr, Araújo OD, Macêdo SF, Silva GVD, Lopes WMPS, et al(2020)^(19)^	Brasil	Transversal	Atenção básica	*Screening of Activity Limitation and Safety Awareness (SALSA) scale e participation scale.*
Achdiat PA, Ariyanto EF, Simanjuntak MN (2021)^(8)^	Indonésia	Revisão	^*^	^*^
Sousa NP, Silva MIB, Lobo CG, Barboza MCC, Abdon APV (2011)^(5)^	Brasil	Transversal	Atenção especializada	*Medical Outcome Study 36-item Short-form health* *Survey (SF36)*; questionário abordando aspectos epidemiológicos, socioeconômicos e característica da doença.
Carvalho MAJ, Lopes NTB, Santos TS, Santos KS, Farnocchi PG, Tavares CM (2013)^(39)^	Brasil	Descritivo	Atenção básica	Questionário Sociodemográfico; Formulário para Avaliação Neurológica Simplificada.
Benedicto CB, Marques T, Milano AP, Galan NGA, Nardi ST, Duerksen Frank, et al (2017)^(20)^	Brasil	Descritivo	Atenção especializada	Formulário de Avaliação do Grau de Incapacidade; Questionário sociodemográfico e clínico; Desenho da Figura Humana (DFH); *NeuroQol - Neuropathy - Specific* *Qualityof Life Questionnaire* (Versão Português Brasileiro).
Govindasamy K, Jacob I, Solomon RM, Darlong J(2021)^(38)^	Índia	Transversal	Atenção básica	*Standardized Questionnaire for depression-(PHQ-9); Standardized questionnaire for Anxiety Disorder-(GAD-7)*; Grau de incapacidade; e ,perfilsocioeconomico
Ekeke N, Chukwu J, Nwafor C, Ogbudebe C, Oshi D, Meka A, Madichie N (2014)^(34)^	Nigéria	Descritivo-retrospectivo, transversal	Atenção especializada	*Questionnaire-based interview; A retrospective descriptive desk analysis of leprosy case notification data for children 0 to 14 years in the 14 states in Southern Nigeria*
Seshadri D, Khaitan BK, Khanna N, Sagar R (2015)^(43)^	Índia	Transversal	Atenção hospitalar	Dados clínicos e demográficos; *52-item AnandarajDehabilitationscale*
Van Dorst MMAR, Van Netten WJ, Waltz MM, Pandey BD, Choudhary R, Van Brakel WH (2020)^(35)^	Nepal	Transversal	Atenção básica	Questionário sociodemografico; *Patient Health Questionnaire (PHQ-9); Warwick-* *Edinburgh Mental Wellbeing Scale (WEMWBS);5-Question Stigma Indicator-Affected Persons (5-QSI-AP)*
Van Brakel WH, Sihombing B, Djarir H, Beise K, Kusumawardhani L, Yulihane R, et al (2020)^(21)^	Indonésia	Transversal	Atenção básica	*Screening of Activity Limitation and Safety Awareness (SALSA) scale, Participation* *Scale, Jacoby Stigma Scale (anticipated stigma), Explanatory Model Interview Catalogue (EMIC) stigma scale* *And Discrimination assessment.*
Beltrame RT, Marciano LHSC, Fonseca MDS; Prado RBR (2015)^(22)^	Brasil	Descritivo	Atenção esoecializada	Questionário sociodemográficos, aspectos clínicos da doença e a independência funcional na execução das AVDs e AIVDs; checklist relacionado ao tipo de deficiência física visível encontrada no paciente; e o Inventário de Estratégias de Coping de Folkman e Lazarus, adaptado para o português
Viana LDS, Aguiar MIFD, Vasconcelos PFD, Aquino DMCD (2017)^(23)^	Brasil	Descritivo, com abordagem quantitativa	Atenção especializada	Domínio Físico do *World Health Organization quality of life (WHOQOL-BREF)* e as Facetas “Habilidades Sensoriais” e “Autonomia” do WHOQOL - OLD
Van Veen NHJ, Hemo DA, Bowers RL, Pahan D, Negrini JF, Velema JP, et al (2011)^(24)^	Bangladesh	Cohort prospectiva	Atenção hospitalar	*Screening of Activity Limitation and Safety Awareness (SALSA) scale, Participation* *Scale*
Hurtado MN, Atehortua MC, Bravo, J (2003)^(25)^	Colômbia	Descritivo	Atenção básica	Classificação Internacional de Funcionalidade; formulário sociodemográfico
Sharma M, Saxena V (2019)^(26)^	Índia	Transversal, observacional	Atenção especializada	*Dados demográficos socioeconômico e epidemiológico;* *and* *availability of various government and nongovernment* *facilities;Body Mass Index - BMI;WHO Disability Grading*
Vieira CSCA, Lobato ML, Figueira MCS, Amaral MCE, Vilela MFG, Silva EM (2018)^(27)^	Brasil	Ecológico, analítico	Atenção básica	Avaliação neurológica simplificada; questionário sociodemográfico e perfil clínico; Screening of Activity Limitation and Safety (SALSA); escala de participação
Nardi SMT, Paschoal VD, Zanetta DMT (2012)^(28)^	Brasil	Transversal	Atenção especializada	*Screening of Activity Limitation and* *Safety Awareness (SALSA) scale*
Monteiro LD, Alencar CH, Barbosa JC, Novaes CCBS, Silva RCP, HeukelbachI J (2014)^(29)^	Brasil	Transversal	Atenção básica	Avaliação neurológica simplificada; *Screening of Activity Limitation and Safety Awarenes (SALSA)*; Escala de participação
D’Azevedo SSP, et al (2021)^(30)^	Brasil	Transversal	Atenção básica	Questionário sociodemográfico e clínico e o instrumento *World Health Organization Disability Assessment Schedule (WHODAS)*
Reis FJJ, Lopes D, Gosling AD, Gomes MK (2014)^(41)^	Brasil	Transversal	Atenção hospitalar	*Douleur* *neuropathique en 4 questions questionnaire; 12-item General Health Questionnaire; World Health Organization quality of life (WHOQOL-BREF)*
Govindharaj P, Srinivasan S, Darlong J (2018)^(9)^	Índia	Transversal descritivo	Atenção hospitalar	Questionário semiestruturado
Ramos JM, Alonso-Castaneda B, Eshetu D, Lemma D, Reyes F, Belinchon I, et al (2014)^(44)^	Etiópia	Transversal	Atenção especializada	*Neuropathic Pain Symptom Inventory (NPSI) questionnaire*
Rodini FCB, Gonçalves M, Barros ARSB, Mazzer N, Elui LMC, Fonseca MCR (2010)^(36)^	Brasil	Transversal	Atenção especializada	*Medical Outcome Study 36-item Short-form health* *Survey (SF36)*
Patil A, Mayur SS (2021)^(42)^	Índia	Transversal	Atenção hospitalar	*World Health Organization quality of life (WHOQOL-BREF); Questionnaire and mental health status by the SelfReporting questionnaire*
Gaudenci EM, Nardelli GG, Almeida Neto OP, Malaquias BSS Carvalho BT, Pedrosa LAK (2015)^(40)^	Brasil	Transversal, quantitativo, descritivo e analítico	Atenção especializada	Questionário clínico e socioeconômico; Inventário de depressão de Beck- BDI para avaliação dos graus de depressão; *World Health Organization quality of life (WHOQOL-BREF)*
Costa RMPC, Mendes LCB(2020)^(10)^	Brasil	Revisão integrativa	^*^	^*^
Govindharaj P, Srinivasan S, Darlong J(2018)^(45)^	India	Transversal	Atenção hospitalar	*World Health Organization quality of life (WHOQOL-BREF)*
Lustosa AA, Nogueira LT, Pedrosa JIS, Teles JBM, Campelo V (2011)^(46)^	Brasil	Transversal	Atenção especializada	*Medical Outcome Study 36-item Short-form health* *Survey (SF36),* dados clínicos, sociodemográficos e epidemiológicos
Chingu D, Duncan M, Amosun S (2013)^(47)^	Malawi	Transversal	Atenção básica	*World Health Organization quality of life (WHOQOL-BREF)*
Schuller I, Van Brakel WH, Van Vliet I, Beise K, Wardhani L, Silwana S, et al (2010)^(37)^	Indonésia	Quantitativo-qualitativo	Atenção básica	Entrevistas e grupos focais
Prado GD, Prado RBR, Marciano LHSC, Nardi SMT, Cordeiro JA, Monteiro HL (2011)^(31)^	Brasil	Transversal	Atenção especializada	*World Health Organization degree* *of physical disability classification (WHO-DG), the International Physical ActivityQuestionnaire (IPAQ) and the Medical Outcome Study 36-item Short-form health* *Survey (SF36)*
Gonçalves M, Prado MAR, Silva SS, Santos KS, Araujo PN, Fortuna CM (2018)^(32)^	Brasil	Qualitativo	Atenção especializada	Entrevistas semiestruturadas
Barcelos RMFM, Sousa GS, Almeida MV, Palacio FGL, Gaíva MAM, Ferreira SMB^(7)^	Brasil	Revisão	^*^	^*^

* *Por se tratar de revisão da literatura não há um cenário e o instrumento é o próprio método.*

## DISCUSSÃO

Os estudos incluídos nesta revisão possibilitaram identificar os impactos da incapacidade física causada pela hanseníase na qualidade de vida dos indivíduos atendidos na Rede de Atenção à Saúde. O Brasil^(5,7,10,17-23,27-32,36,40-41,46)^ foi o país que mais publicou e demonstrou sua importância epidemiológica, com predomínio dos estudos transversais. Os estudos incluídos nesta revisão demonstraram os seguintes impactos desencadeados pela doença:

- impacto nas atividades de vida diária^(5,17-18,20-32)^;
- impacto socioeconômico^(19-21,24,26-37,39)^;
- impacto psicológico^(5,8,22,35,38-43)^;
- impacto da dor^(15,32,36,41,44)^;
- impacto no bem-estar geral^(7,10,23,40-41,45-47)^.

Ao se considerar o impacto da limitação nas atividades de vida diária, observaram-se prejuízos no aspecto físico, o que leva a piores níveis de qualidade de vida dessa população. As limitações no aspecto físico incluem dor, mobilidade reduzida, deformidades que impactam negativamente nas atividades de vida diária como autocuidado, deslocamento, alimentação, higiene pessoal, vestir, despir e calçar. Além disso, outras atividades cotidianas como gerir a própria casa, realizar compras e utilizar transportes, são prejudicadas e interferem na integração social^(17-18,21-22,28-29)^.

Os estudos retrataram uma forte relação entre o GIF 1 e 2, a forma multibacilar e o grau de dependência nas Atividades Básicas de Vida Diária (ABVD) e Atividades Instrumentais de Vida Diária (AIVD)^(15-17,21-24,26-28,30-31)^. Foi observada alta prevalência de incapacidade física, com amputações e deformidades visíveis na população idosa^(15)^. Destaca-se ainda que mesmo após a alta por cura da doença, há casos em que os impactos são permanentes^(26)^. Por outro lado, verificou-se que a cirurgia reconstrutiva pode ser eficaz na melhora das atividades de vida diária. Portanto, é uma estratégia para melhorar a qualidade de vida dessas pessoas^(23)^.

O instrumento específico para a avaliação da limitação das atividades de vida diária, o SALSA, foi um dos mais utilizados^(17-19,21,24,27-29)^. Nesse sentido, essa escala pode subsidiar os serviços no tratamento, visto que o GIF2 no diagnóstico indica detecção tardia e fatores intervenientes como dificuldades operacionais dos serviços, desinformação sobre sinais e sintomas, dificuldade de acesso aos serviços e a profissionais capacitados^(29)^.

Ao se tratar do impacto dos aspectos sociais e econômicos, foi verificado que a maior parte da população acometida é do sexo masculino, faixa etária acima de 40 anos, com baixa escolaridade, casados ou viúvos, desempregados ou sem ocupação e baixa renda familiar^(21,26,39,44-45)^. Portanto, indica a relação do GIF atrelado aos aspectos sociais e econômicos, o que reporta para baixo nível nos escores de qualidade de vida^(44)^. Ao passo que a hanseníase continua acometendo pessoas mais vulneráveis aos determinantes socioeconômicos, as intervenções dos profissionais de saúde em relação à educação em saúde e orientação sobre autocuidado podem repercutir positivamente na qualidade de vida dos portadores da doença^(9)^.

Nessa direção, estudo realizado com 26 pessoas acometidas pela hanseníase avaliou a qualidade de vida desse grupo antes e após um ano da implementação de um guia de autocuidado. Os autores identificaram melhora na dor, na função motora, nas condições da pele e aspectos sociais^(36)^. Em relação aos aspectos sociais, a restrição à participação social tem sido associada a limitações nas questões de aprendizado e aplicação dos conhecimentos, na comunicação, na mobilidade, no autocuidado, nos relacionamentos interpessoais e na vida comunitária. Os estudos incluídos na revisão que aplicaram essa escala constataram altos índices de restrição à participação social^(18-19,24,27-28,33)^.

Ao retratar os impactos dos aspectos psicológicos, o estigma e o autoestigma causados pela doença geram consequências sociais e emocionais negativas, que podem acarretar em elevados índices de distanciamento social na comunidade^(8)^. Esses casos são mais frequentes em pessoas com GIF2, menor renda familiar e grau de escolaridade mais baixo. Com relação ao gênero, as mulheres sofrem com a estigmatização em maior proporção devido às restrições à participação social e menores perspectivas de matrimônio^(21,33,37,40)^. O padrão estético idealizado frequentemente é mais intenso em relação às mulheres, o que estabelece um nível mais elevado de estigma relacionado à doença^(20,32)^.

Para o enfrentamento do estigma, conhecer a vivência do paciente acometido pela hanseníase pode possibilitar o planejamento de estratégias dos serviços de saúde. Com esse objetivo, podem ser utilizadas a Escala de Estigma para Pessoas Acometidas pela Hanseníase (EMIC-AP) e a Escala de Participação, conforme recomendado no Protocolo Clínico e Diretrizes Terapêuticas da Hanseníase (PCDT hanseníase)^(48)^. Além disso, essas escalas podem contribuir para a melhoria da qualidade da atenção, promovendo um cuidado integral, além de direcionar para ações na atenção psicossocial e saúde mental^(48)^.

Os baixos níveis de bem-estar psíquico e altos níveis de depressão são mais frequentes nas pessoas com incapacidade física pela hanseníase quando comparados com a população em geral. Isso se deve ao grande estigma da doença que intensifica os sentimentos como ansiedade, tristeza, medo, baixa autoestima, distúrbios do sono e até mesmo pensamentos suicidas. Desse modo, muitos acometidos sentem culpa e vergonha por terem contraído a doença, o que leva a prejuízos nas relações afetivas^(8,20,29,34,)^. A psicoterapia pode favorecer a compreensão da condição física e emocional, além da reorganização da imagem corporal e melhorar a autoestima^(5)^.

A utilização de estratégias como educação em saúde e manuais de orientação contribuem para o aumento do nível de instrução, esclarecimento sobre o tema e acesso a um tratamento multidisciplinar adequado^(36,49)^. Entende-se que programas de intervenções multidisciplinares para educação em saúde envolvem intervenções de cuidado holístico, saúde física, mental e psicossocial para a comunidade e familiares dos doentes, consequentemente são capazes de melhorar os índices de qualidade de vida^(36)^.

Nesse sentido, a avaliação da evolução da doença de forma contínua e humanizada e a realização da busca ativa de casos novos são ferramentas indispensáveis na prevenção de agravamentos, bem como na melhora do bem-estar psicossocial^(35)^. No impacto da dor, destaca-se a neuropática, reconhecida como outra complicação tardia em pacientes com hanseníase.

Evidencia-se alta prevalência da dor referida, o que leva a significativas alterações do sono e de atividades da vida diária^(42)^, associada ao grau de incapacidade física, à participação social^(27-28)^ e ao sofrimento psicológico^(36)^. O dano progressivo nos nervos periféricos pode levar a um estado crônico da dor, mesmo após o fim do tratamento^(41)^. A dor crônica está associada ao sofrimento psicológico e é um importante preditor de má qualidade de vida. Portanto, o manejo da dor pode contribuir para redução das desordens psicológicas e melhora na qualidade de vida^(41)^.

Na avaliação da qualidade de vida, os domínios que apresentaram piores escores foram limitação por aspecto físico, dor e aspecto emocional^(17)^. Foi verificado que o maior comprometimento esteve relacionado à perda/redução da sensibilidade^(5)^ com predominância das formas incapacitantes da hanseníase^(21)^. As incapacidades físicas pela hanseníase muitas vezes não são reversíveis, no entanto é possível obter qualidade de vida quando ações de prevenção são estabelecidas.

Nesse sentido, estudo^(36)^ utilizou um manual para prevenção de incapacidades, repercutindo na melhora da qualidade de vida nos domínios dor e aspectos sociais do questionário SF-36. Além disso, detectaram que a dor, o desconforto e a dependência de medicamentos estiveram relacionados com maiores implicações no comprometidos do domínio físico^(36)^. Outros estudos apontam que o domínio físico foi o mais impactado, com relação inversamente proporcional à GIF, ou seja, quanto maior o GIF menor os escores de qualidade de vida^(36,44)^. Em decorrência disso, notou-se a diminuição das atividades laborais e restrição à vida social o que gera prejuízos à qualidade de vida^(17,25-26,40,47)^.

O termo Qualidade de Vida Relacionada à Saúde (QVRS) é mais específico e envolve o impacto de uma doença na qualidade de vida^(10)^. Foi verificado que quanto maior o grau de evolução da doença e o estabelecimento das formas incapacitantes, pior a QVRS. A fim de analisar a qualidade de vida de pacientes com hanseníase, identificaram cinco determinantes da QVRS: diagnóstico tardio, formas multibacilar, reações, GIF2 no diagnóstico e preconceitos^(10)^.

Na presente revisão houve predominância de estudos realizados na atenção especializada e, consequentemente, maiores foram os impactos das incapacidades físicas. Na avaliação da qualidade de vida e das limitações de atividades, identificou-se a aplicação dos instrumentos *WHOQOL-bref* ^(10,30,36,42,46)^, *SF-36* ^(5,31,36,46)^ e o *SALSA* ^(18-19,21,24,29)^ na Atenção Básica, Especializada e Hospitalar, conforme documento suplementar

Cabe ressaltar a representatividade da enfermagem na Rede de Atenção à Saúde no Brasil. Ao enfermeiro, enquanto responsável pela equipe de enfermagem, cabe planejar e prestar esclarecimento acerca da hanseníase, prevenção de incapacidades, autocuidado, tratamento, bem como realizar atividades de busca ativa de novos casos. Assim, a consulta de enfermagem se torna essencial no estabelecimento do vínculo entre enfermeiro e a pessoa com hanseníase^(50)^.

Por fim, a capacitação dos profissionais da saúde, em especial da enfermagem, atividades de educação em saúde, definição de atribuições profissionais e uso de instrumentos específicos na assistência de enfermagem podem contribuir para uma assistência efetiva e uma melhora da qualidade de vida dos pacientes. Sugere-se que durante as consultas de enfermagem sejam aplicadas escalas como o *WHOQOL-bref, SF-36* e o *SALSA*, bem como as indicadas no PCDT hanseníase (a EMIC-AP e escala de participação)^(48)^, pois podem subsidiar ações para o cuidado de enfermagem.

### Limitações do estudo

Reconhecemos algumas limitações inerentes a esta revisão. A primeira delas refere-se à seleção de bases de dados, que, embora abrangente, pode ter deixado de incluir possíveis contribuições de outras fontes. Além disso, a predominância de estudos realizados no Brasil pode limitar a generalização dos resultados para contextos diferentes, destacando a necessidade de mais pesquisas em outros países. A heterogeneidade nos métodos e instrumentos de avaliação utilizados nos estudos incluídos pode também introduzir variações nos resultados, dificultando uma síntese mais uniforme das evidências.

Por fim, a ausência de uma análise detalhada da qualidade metodológica dos estudos incluídos pode afetar a confiabilidade dos resultados apresentados. Essas considerações oferecem uma perspectiva crítica sobre as conclusões derivadas desta revisão, destacando a importância de futuras pesquisas para superar essas limitações e enriquecer nossa compreensão dos impactos da hanseníase na qualidade de vida.

### Contribuições do estudo para a área da Enfermagem

As evidências geradas a partir deste trabalho desempenham um papel significativo na atenuação dos impactos gerados pela hanseníase na qualidade de vida das pessoas, proporcionando benefícios tangíveis para a assistência à saúde nas áreas de prevenção, promoção e reabilitação. Ao oferecer uma reflexão abrangente sobre os aspectos envolvidos na assistência integral ao indivíduo, o estudo promove a articulação do debate entre os diversos atores envolvidos no planejamento das políticas de saúde, visando aprimorar as ações de controle da hanseníase.

## CONCLUSÕES

Nosso estudo permitiu identificar e sintetizar os impactos da incapacidade física causada pela hanseníase e suas implicações na qualidade de vida dos indivíduos atendidos na Rede de Atenção à Saúde. Assim, os impactos da hanseníase na qualidade de vida influenciaram as atividades de vida diária, as atividades sociais e econômicas, a saúde mental, o bem-estar e o nível de dor.

O mapeamento da literatura mostrou a dimensão do tema e a importância da necessidade de ações de controle da doença, como o diagnóstico precoce e oportuno, fundamental para evitar o desenvolvimento da incapacidade física, responsável por desencadear inúmeras complicações que prejudicam diretamente a qualidade de vida. Portanto, são imprescindíveis as ações de prevenção, promoção e reabilitação, além da educação permanente dos profissionais de saúde, para o rastreamento e o acompanhamento dos casos.

O enfermeiro, enquanto membro da equipe multiprofissional, desempenha um papel fundamental no cuidado a essa população por meio do esclarecimento sobre a doença, da prevenção de incapacidades, do autocuidado, do tratamento, bem como na busca ativa de novos casos. Cabe destacar que, além do conhecimento clínico, a utilização de instrumentos de medida pode qualificar a assistência a essa população.
